# Construction of an Integration Vector with a Chimeric Signal Peptide for the Expression of Monoclonal Antibodies in Mammalian Cells

**DOI:** 10.3390/cimb46120868

**Published:** 2024-12-22

**Authors:** Valentina S. Nesmeyanova, Daniil V. Shanshin, Denis E. Murashkin, Dmitriy N. Shcherbakov

**Affiliations:** State Scientific Center of Virology and Biotechnology “Vector”, Rospotrebnadzor, 630559 Koltsovo, Novosibirsk Region, Russia; nesmeyanova_vs@vector.nsc.ru (V.S.N.); dan6091154224@gmail.com (D.V.S.); denmurashkin@gmail.com (D.E.M.)

**Keywords:** artificial signal sequence, integrative vectors, recombinant antibodies, CHO-K1

## Abstract

Antibodies are complex protein structures, and producing them using eukaryotic expression systems presents significant challenges. One frequently overlooked aspect of expression vectors is the nucleotide sequence encoding the signal peptide, which plays a pivotal role in facilitating the secretion of recombinant proteins. This study presents the development of an integrative vector, pVEAL3, for expressing full-length recombinant monoclonal antibodies in mammalian cells. The vector features a distinctive nucleotide sequence that encodes an artificial chimeric signal peptide with the following amino acid sequence: MMRTLILAVLLVYFCATVHC. Additionally, the vector incorporates several regulatory elements to enhance antibody expression, including the Gaussia luciferase signal sequence, internal ribosome entry site (IRES), P2A peptide, and a furin cleavage site. These elements coordinate to regulate the synthesis levels of the antibody chains. The analysis of clones obtained via transfection with the developed vector showed that over 95% of them secreted antibodies at levels significantly higher than those of the control. The immunochemical analysis of the chimeric antibody produced by the CHO-K1-10H10ch cell line confirmed the preservation of its functional activity.

## 1. Introduction

Antibodies are complex protein assemblies, and a functional antibody consists of a tetrameric structure made up of two heterodimers. The production of such proteins using eukaryotic expression systems is a challenging task. An optimal solution to this problem is the creation of an expression cassette containing the most suitable genetic elements, ensuring the synthesis and secretion of the target protein in the producer cells [1]. Vector design is a key methodology for enhancing recombinant antibody synthesis under stable expression conditions [2]. Transcription in mammalian cells is a complex process regulated by numerous proteins. Therefore, it is essential to include both primary and auxiliary genetic elements in the expression cassette, arranged in the correct orientation [3]. While promoters play a crucial role, the combination of regulatory elements and the prevention of target gene silencing can significantly impact the expression and stability of recombinant proteins [3].

One of the principal challenges in producing functional antibodies is ensuring the balanced expression of the light and heavy chains, as well as their correct tetramerization. The initial strategy to address these challenges involved the use of a dual-plasmid system, where each plasmid encoded either the heavy or light chain. This approach is now a standard methodology for the expression of recombinant antibodies in research, as it avoids the need for complex vector design [4]. The main disadvantage of this approach is that the integration of the light and heavy chain genes into the chromosomes exhibits variability, both in terms of insertion site and copy number. This variability results in an imbalance in the expression levels of the light and heavy chains, with the light chain often being expressed at higher levels than the heavy chain [5].

An alternative approach involves the independent expression of genes encoding the light and heavy chains within a single vector. This method uses distinct promoters and sequences to ensure independent polyadenylation. In theory, this strategy should result in equal expression levels for both the light and heavy chain genes. However, there is a risk of transcriptional interference, which could lead to an imbalance in transcription and, consequently, inhibit recombinant antibody synthesis [2]. The identification of elements such as internal ribosome entry sites (IRESs) and 2A peptides has facilitated the development of polycistronic vectors. These constructs allow the genes encoding the light and heavy chains of the target antibody to be controlled by a single promoter in a single reading frame [6,7,8]. The pivotal role in such constructs is played by elements that facilitate the coordinated expression of multiple subunits from a single mRNA. One of the most extensively researched components is the IRES, a highly structured RNA sequence that regulates the initiation of mRNA translation through a cap-independent mechanism [9,10,11,12]. The IRES element was initially identified in the viral RNA of *poliovirus* and has since been found in other virus families, including *Flaviviridae*, *Retroviridae*, and *Herpesviridae* [10,12]. IRES elements have also been identified in various contexts, including both viral RNAs and cellular mRNAs [12,13]. 2A peptides are another important tool for achieving coordinated expression. These short viral amino acid sequences, comprising 18 to 25 residues, induce ribosomal skipping during translation [14,15,16]. This mechanism, also referred to as the “stop-carry on” process, facilitates the co-expression of multiple genes from a single transcript [14,17,18,19]. 2A peptides are widely distributed in viruses affecting insects and mammals [16,17]. The four key 2A peptides identified to date (F2A, E2A, P2A, and T2A) have been widely used for co-expressing proteins in genetic constructs. These sequences offer a flexible approach to synthesizing multiple proteins under the control of a single promoter, providing a solution to the limitations of traditional methods such as IRESs and dual-promoter systems [16,17]. Consequently, the use of tools like IRESs and 2A peptides has significantly improved the process of monoclonal antibody production, enabling large-scale production with the desired characteristics and biological activity [20,21,22].

It is also worth noting that, in addition to the aforementioned tools, signaling peptides play an essential role in the efficient production of recombinant proteins. Signal peptides are crucial for the synthesis and secretion of recombinant proteins, guiding the transport of the nascent polypeptide to the lumen of a subcellular compartment and initiating the protein secretion pathway. However, natural signal peptides are not always optimal for efficient expression in heterologous systems. Therefore, it is essential to develop new signal peptides to enhance recombinant protein production in these systems [3,23,24,25].

The objective of this study was to develop an integrative vector to facilitate the production of recombinant monoclonal antibodies in mammalian cells. To achieve this, specific elements, including signal peptides 176 and GL, an internal ribosome entry site (IRES), and the P2A peptide, were employed.

## 2. Materials and Methods

### 2.1. Plasmid Vectors and Cell Cultures

The plasmid vectors pVEAL3-10H10ch and pSB100X were used in this study. The adherent CHO-K1 cell culture (Chinese hamster ovary) was obtained from the cell culture collection of the Federal Budgetary Institution of Science, “State Research Center of Virology and Biotechnology ‘Vector’”. The Escherichia coli strain (NEB Stable F’ proA+B+ lacIq ∆(lacZ)M15 zzf: Tn10 (TetR) ∆(ara-leu) 7697 araD139 fhuA ∆lacX74 galK16 galE15 e14- Φ80dlacZ∆M15 recA1 relA1 endA1 nupG rpsL (StrR) rph spoT1 ∆(mrr-hsdRMS-mcrBC)) was provided by Invitrogen (USA).

### 2.2. Construction of the Integration Vector pVEAL3-10H10ch

The variable sequence of the gene encoding the 10H10 heavy chain (GenBank OK483332) and the variable sequence of the gene encoding the 10H10 light chain (GenBank OL448869) were retrieved from the GenBank database and optimized for codon composition for CHO cells using the online Codon Optimisation Tool software (https://www.idtdna.com/pages/tools/codon-optimization-tool, accessed on 3 December 2024). The final nucleotide sequence was supplemented with sequences encoding the 176 signal peptide, the constant region of the light chain, the cleavage site for cellular protease furin, the self-cleaving P2A peptide, the Gaussia luciferase signal sequence, and the constant region of the heavy chain (CH1-CH2-CH3), as well as restriction sites for NheI and BstXI. The synthesized nucleotide sequence of 10H10 was cloned into the pVEAL3 vector using standard cloning techniques [26]. Polymerase chain reaction (PCR) was used to amplify the synthesized nucleotide sequence with Q5 High-Fidelity DNA polymerase. The primers 10H10-pVL3_F (5′-aaaaaaaaagctagCACCACCATGATGCGGACCCTGATCCTGGCTGTGCTGCTGCTG-3′) and 10H10-BstXI_R (5′-aaaaaaGTGCAGCTGCTGCTGGAGGGCACGG-3′) were used for amplification. The PCR products and plasmid vector pVEAL3 were then treated with NheI and BstXI restriction endonucleases and purified using the Gel Extraction Kit (Qiagen, Hilden, Germany), according to the manufacturer’s instructions. The ligation of the PCR product with the pVEAL3 vector was performed using T4 DNA ligase (SibEnzyme, Novosibirsk, Russia). The resulting constructs were introduced into competent *Escherichia coli* cells (NebStable strain) by transformation. The primary structure of the expression vector was verified by Sanger sequencing at the Genomics Centre of the Siberian Branch of the Russian Academy of Sciences (Novosibirsk).

### 2.3. Generation of the Producer Strain CHO-K1-10H10ch

The CHO-K1 cell line stably expressing full-length recombinant 10H10 antibody was obtained by transfection with the integrative plasmid vector pVEAL3-10H10ch. The cells were cultured in an incubator set to 37 °C, 5% CO_2_ (Heracell VIOS 160i, 165 L, Thermo Fisher Scientific, Waltham, MA, USA), with 80% relative humidity. Transfection was performed at 80% monolayer density using the Lipofectamine 3000 reagent (Invitrogen, Carlsbad, CA, USA), according to the manufacturer’s instructions. A 1:10 mixture of pVEAL3-10H10ch and pSB100X plasmids was introduced into the cells. After 48 h, the selective antibiotic puromycin (Servicebio, Wuhan, China) was added to the culture medium at a concentration of 10 μg/mL. Over the next three days, the majority of cells were observed to die. On the fourth day, the surviving cells were transferred to 6-well culture plates, which were supplemented with the selective antibiotic. After two days, a dense cell monolayer was established, representing a polyclonal pool. The limiting dilution method was used to obtain highly productive monoclonal lines. The cell culture was diluted to 1–0.5 cells/mL by serial dilution in F12/DMEM medium (PanEco, Moscow, Russia) supplemented with 10% FBS (Gibco, Thermo Fisher Scientific, USA) and 2 mM GlutaMAX (Gibco, Thermo Fisher Scientific, USA). The resulting cell suspension was transferred in 200 μL aliquots into the wells of a 96-well plate using a multichannel pipette and incubated at 37 °C, 5% CO_2_ for 14 days. Antibody production in the supernatants was analyzed using enzyme-linked immunosorbent assay (ELISA). The clone with the highest antibody production, designated 10H10, was selected for subsequent large-scale culturing using the roller bottle system.

### 2.4. Roller Cultivation of the CHO-K1-10H10ch Producer Strain

For seeding cells in roller bottles, DMEM/F12 medium (PanEco, Russia) supplemented with 4 mM GlutaMax (Gibco, Thermo Fisher Scientific, USA) and 10% FBS (Gibco, Thermo Fisher Scientific, USA) was prepared at a volume of 200 mL per roller bottle (Jet Biofil, Guangzhou, China). The roller bottles were then inoculated with CHO-K1-10H10ch producer strain cells at a density of 1 × 10^5^ cells/mL. The bottles were transferred to a roller apparatus and incubated at 37 °C for 7–10 days, with a rotation speed of 0.5–1 rpm. After the formation of a monolayer, the growth medium was replaced with maintenance medium (DMEM/F12 with 2% FBS), and the cells were cultured for an additional week. To subculture cells from one roller bottle to another, the culture supernatant was decanted, and 25 mL of TrypLE™ Express Enzyme (Thermo Fisher Scientific, USA) was added, followed by incubation at 37 °C on the roller apparatus for 4 min. Once the cells were completely detached from the substrate, they were thoroughly dispersed. The cell count was then determined using the TC20 cell counter (Bio-Rad, California, CA, USA). Fresh growth medium (200 mL) was added to a new roller bottle, and the cells were seeded at a density of 1 × 10^5^ cells/mL.

### 2.5. Affinity Purification of Chimeric Full-Length Antibody 10H10ch

The culture medium containing the antibodies was centrifuged for 10 min at 12,000× *g* and filtered through 0.22 µm pore-size filtration systems to remove cellular debris. Antibody purification was performed using a sorbent with Protein A on its surface (MabSelect SuRe, GE Healthcare, Chicago, IL, USA).

The equilibration of the chromatography column for antibody purification involved washing with five column volumes of the base buffer (50 mM Tris-HCl, 150 mM NaCl, pH 7.0–7.2). The flow rate was adjusted based on the buffer contact time with the sorbent, with a minimum contact time of 4 min. Samples were then loaded onto the column, ensuring a sample contact time of at least 5 min. The column was washed to remove unbound proteins and those with a low binding affinity through a sequential washing protocol. Initially, the column was washed with 3.5 column volumes of the base buffer, followed by treatment with 3.5 column volumes of the wash buffer (20 mM Tris-HCl, pH 6.8–7.0). The flow rate during the washing steps was set to ensure a buffer contact time of at least 4 min. Antibodies bound to the column were eluted using an elution buffer (100 mM glycine-HCl, pH 2.7–2.8). The eluted fractions were immediately neutralized with 1 M Tris-HCl (pH 9.0) to achieve a final pH of 7.0.

### 2.6. Purification of Mouse 10H10 Antibody with Caprylic Acid from Ascites Fluid

Monoclonal antibodies (MCAs) were purified using caprylic acid and ammonium sulfate. The ascitic fluid or serum was centrifuged at 5000× *g* for 5–10 min to remove larger cellular components and obtain a clarified supernatant. The samples were diluted with 1 × acetate buffer (pH 4.0) at a 1:4 ratio (sample:buffer). After confirming and adjusting the pH to 4.0, 25 μL of caprylic acid was added to each 1 mL of sample with constant stirring. The mixture was incubated at room temperature for 30 min, either with continuous stirring or in a shaking incubator. Alternatively, the mixture was incubated overnight at 4 °C. Following incubation, the samples were centrifuged at 10,000× *g* for 30 min. The supernatant was then filtered through filter paper, and the precipitate was stored for potential protein loss assessment. For further purification, a saturated ammonium sulfate solution (pH 7.5) was added to the supernatant at a 1:1 ratio. The mixture was incubated at 4 °C for 1–3 days to precipitate the immunoglobulins. After the incubation period, the precipitated protein was isolated by centrifugation at 10,000× *g* for 30 min and then dissolved in PBS buffer.

### 2.7. Enzyme-Linked Immunosorbent Assay (ELISA)

Recombinant fragments of envelope proteins from tick-borne encephalitis virus (TBEV), Zika virus (ZIKV), dengue virus (DENV), and West Nile virus (WNV) (designated TEF1, ZEF1, DEF1, and WEF1, respectively), representing recombinant analogs of domains 1 and 2 of the E protein [16], were used as antigens in the ELISA at a concentration of 200 ng/well. Antigens were coated in 96-well plates (Greiner bio-one, Frickenhausen, Germany) in phosphate-buffered saline (PBS) at 4 °C overnight. The wells were washed with PBST buffer (PBS containing 0.1% Tween-20) and blocked with 1% casein in PBST for 1 h at room temperature. Diluted samples (1:100) were added and incubated for 1 h at 37 °C. For the recombinant chimeric antibody 10H10ch, a Goat anti-Human IgG-peroxidase conjugate (Sigma-Aldrich, St. Louis, MO, USA, 1:5000) was used, while for the native antibody (positive control), a Goat anti-Mouse IgG-peroxidase conjugate (Sigma, 1:5000) diluted in a 1% casein solution was added. Each step was followed by washing with PBST. The substrate solution (TMB, Sigma, USA) was added and incubated for 20 min at room temperature in the dark. The reaction was stopped with 50 μL of 1 M sulfuric acid per well, and optical density (OD) was measured at 450 nm using a microplate reader (Thermo Fisher Scientific, USA).

### 2.8. Statistical Analysis

Statistical analyses were conducted using the GraphPad Prism software (version 9.3.1; GraphPad Software, San Diego, CA, USA). Differences were considered statistically significant at *p* < 0.05.

## 3. Results and Discussion

### 3.1. Design and Construction of the Integration Vector pVEAL3-10H10ch

**Figure 1:**
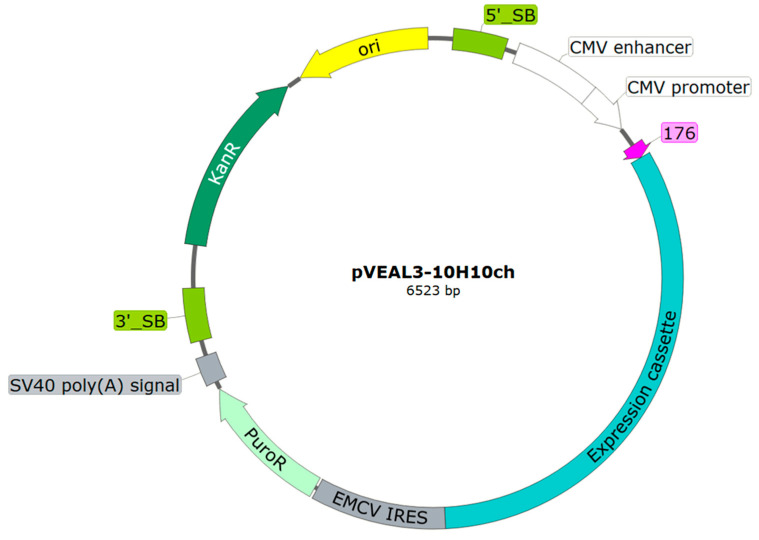
Schematic diagram of the integration plasmid vector pVEAL3-10H10ch. 5′_SB and 3′_SB—transposase SB100X binding sites; CMV promoter—CMV promoter region; 176—nucleotide sequence encoding a hybrid signal peptide from luciferase (*Cypridina noctiluca*) and fibroin (*Dendrolimus spectabilis*), facilitating protein export from the cell; EMCV IRES—internal ribosome entry site; PuroR—nucleotide sequence encoding resistance to the antibiotic puromycin; SV40 poly (A) signal—nucleotide sequence stabilizing mRNA transcripts through polyadenylation; KanR—nucleotide sequence encoding resistance to the antibiotic kanamycin; ori—origin of replication. The complete nucleotide sequence of the developed vector is presented in Figure S1.

The production of full-length antibodies requires the precise control of the expression of both light and heavy chains to ensure the formation of functional tetramers. The primary strategy to address this challenge involves the development of expression cassettes within integrative vectors. Evidence from studies comparing polycistronic and monocistronic vectors highlights the superiority of polycistronic systems in achieving a balanced expression of antibody components, which is critical for the stability and functionality of the final product [27,28,29]. In this study, an integrative vector was designed to incorporate elements that support the synthesis and proper assembly of full-length antibodies (Figure 1).

The integrative vector, pVEAL3-10H10ch, was designed to encode a single open reading frame encompassing both the heavy and light chains of the chimeric antibody. Each chain incorporated the variable regions of the murine 10H10 antibody [30] fused to the constant domains of the human heavy and light chains.

**Figure 2:**
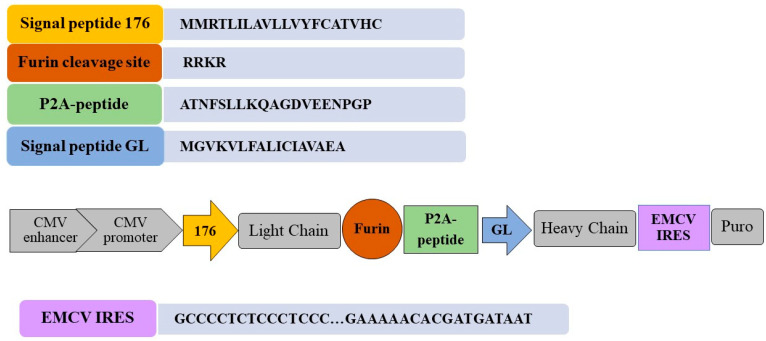
Schematic representation of the expression cassette. Key design elements are highlighted in color. Key elements of the construct: 176—nucleotide sequence encoding a hybrid signal peptide from luciferase (*Cypridina noctiluca*) and fibroin (*Dendrolimus spectabilis*), facilitating protein export from the cell; Furin—nucleotide sequence encoding the proteolytic site for the cellular protease furin; P2A—nucleotide sequence encoding the self-cleaving P2A peptide; GL—nucleotide sequence encoding the Gaussia luciferase signal sequence, facilitating protein export from the cell; EMCV IRES—internal ribosome entry site. Additional elements: CMV promoter—CMV promoter region; Puro—nucleotide sequence encoding the antibiotic resistance factor for puromycin.

To enhance transgene expression and ensure a stable recombinant antibody production during extended cultivation in mammalian cells, the pVEAL3-10H10ch vector included multiple regulatory elements (Figure 2).

A key component of the genetic construct is a strong promoter. Recent studies have demonstrated that the CMV promoter, when paired with a CMV enhancer positioned immediately upstream, increases expression levels by 2–3-fold compared to the CMV promoter alone [31]. To support efficient initiation of protein synthesis, the vector incorporates the consensus Kozak sequence, including the CACC motif preceding the start codon.

When designing expression vectors for the synthesis of complex proteins, it is critical to ensure the synchronous expression of the subunits constituting the protein complex, in addition to achieving highly productive clones. The use of IRES elements, however, can result in variable expression levels of downstream genes within the cassette, ranging from 6% to 100% [17,32]. This variability can be leveraged as a tool for selecting highly productive clones. For example, reducing the downstream expression to 50% can theoretically favor the selection of clones with enhanced target protein production under selective pressure from antibiotics. As reported in the literature, the application of the IRES or its derivatives (e.g., IRES-att) reduces the proportion of non-expressing clones, thereby increasing the overall yield of the target protein [2,33,34].

The design of an expression vector for full-length antibody production requires the precise control of the light-to-heavy chain ratio to ensure the proper assembly of the tetrameric antibody structure. Studies have shown that maintaining equal or slightly higher expression levels of the light chain relative to the heavy chain is critical. To achieve the synchronized expression of both chains, the use of self-cleaving 2A peptides has been widely recommended, as demonstrated in numerous studies [2,6,35]. This strategy enables the coordinated synthesis of light and heavy chains from a single transcript.

However, it is recognized that the cleavage efficiency of 2A peptides may not always be sufficient to achieve optimal system performance [15]. To address this limitation, a furin proteolysis site was incorporated into the construct, enhancing cleavage efficiency and improving overall target protein expression [17]. Additionally, in the developed vector, the sequence encoding the light chain was placed upstream of the heavy chain. This arrangement was designed to enhance antibody expression while minimizing the risk of unwanted oligomerization [36].

**Figure 3:**
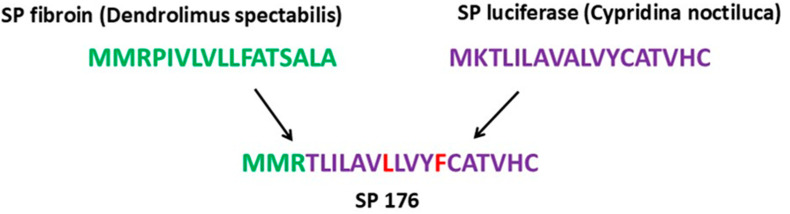
Amino acid sequence of the chimeric signal peptide 176.

A critical component of the developed expression cassette is an artificial signal peptide. The nucleotide sequence 176 encodes a hybrid signal peptide, constructed from fragments of the luciferase signal peptide (derived from *Cypridina noctiluca*) and the fibroin signal peptide (from *Dendrolimus spectabilis*). This hybrid signal peptide, referred to as 176, is theoretically designed to enhance the secretion efficiency of the target protein (Figure 3).

Signal peptide 176 has been previously utilized in the development of the pVAX-RBD plasmid, where it successfully facilitated the secretion of the RBD into the extracellular space [37].

In combination, these elements enable the synthesis of a full-length antibody with confirmed functional activity, as demonstrated by the electrophoresis and ELISA analyses.

### 3.2. Obtaining a Stable Producer Strain CHO-K1-10H10ch

To produce recombinant antibodies, ensuring the completion of key post-translational modifications, such as glycosylation and proper protein folding, is essential. For this reason, mammalian cells have become the preferred production system for recombinant antibodies, particularly full-length variants. Their use enables the generation of biologically active preparations with minimal immunogenicity [38,39].

Among mammalian cell lines, Chinese hamster ovary (CHO) cells account for the production of 84% of therapeutic proteins, the majority of which are recombinant antibodies [40,41]. Consequently, CHO-K1 cells were selected as the production platform for this study.

**Figure 4:**
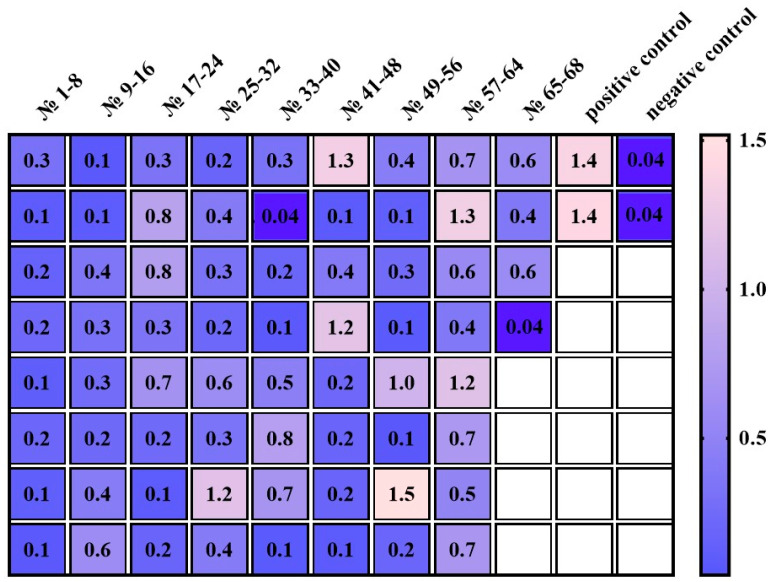
Optical density (OD) values of culture supernatant samples from 10H10ch antibody clones. The positive control, with a murine 10H10 antibody, had an OD of 1.4. The negative control, with a casein protein, had an OD of 0.04.

To assess the efficiency of the developed vector, productivity analyses were conducted on individual clones following transfection into CHO-K1 cells. The Sleeping Beauty transposon system was employed to integrate the expression cassette into the CHO-K1 genome, ensuring stable and long-term transgene expression in producer cells [42,43,44]. Clone selection was carried out using puromycin as the selective agent at a final concentration of 10 µg/mL. The synthesis level of the recombinant chimeric antibody 10H10ch in selected clones was quantified using ELISA (Figure 4).

A full-length murine 10H10 antibody produced via hybridoma technology [45] served as the comparison protein (positive control) in this study. A recombinant fragment of the tick-borne encephalitis virus E protein was used as the antigen. In the ELISA, the murine 10H10 antibody, diluted to 1:1000 (3 μg per well), yielded an optical density (OD) of 1.4. The negative control, the casein protein, produced an OD of 0.04.

Among the 68 monoclonal clones analyzed, only two clones (No. 34 and No. 68) (2.9%) exhibited productivity comparable to the negative control. The OD values of the culture supernatants from the remaining monoclonal clones exceeded the negative control by at least three-fold. Based on these results, seven clones (10%) demonstrated high antibody production with OD values ≥ 1, equivalent to the positive control.

**Figure 5:**
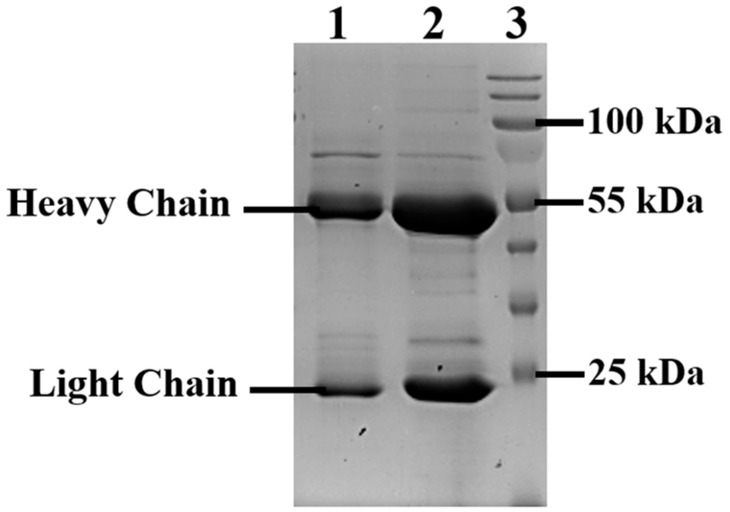
Electrophoresis in 15% SDS-PAGE. Lane 1—recombinant 10H10ch antibody under denaturing conditions (concentration ~10 µg/well); lane 2—murine 10H10 antibody under denaturing conditions (concentration ~20 µg/well); lane 3—molecular weight markers (250–10 kDa). The recombinant 10H10ch antibody was isolated using affinity chromatography. The murine 10H10 was isolated using a caprylic acid purification protocol from mouse ascites fluid.

Clone No. 55 of the CHO-K1-10H10ch producer culture was selected for further work as it exhibited the highest OD value for the interaction between the culture supernatant and the antigen. This clone was gradually scaled up from plate wells to 850 cm^2^ roller culture bottles. Following the cultivation of the CHO-K1-10H10ch producer strain in roller bottles, 1 liter of culture supernatant was harvested. The recombinant antibody was purified via affinity chromatography, yielding 15 mg/L of protein based on the culture supernatant volume. The presence of the target protein was confirmed through electrophoresis under denaturing conditions on a 15% SDS-PAGE gel (Figure 5).

Recombinant 10H10ch and murine 10H10 antibodies showed similar electrophoretic profiles under denaturing conditions, with two major bands corresponding to the heavy chain of 10H10ch at approximately 55 kDa and the light chain at approximately 35 kDa, consistent with the profile of the murine 10H10 antibody. Thus, the presence of key elements in the expression vector enabled the successful production of the full-length recombinant 10H10ch antibody.

The recombinant 10H10ch antibody yield of 15 mg/L, even before any optimization of cultivation conditions, is within the typical range for laboratory-scale production in CHO cells [46]. This yield could potentially be increased by optimizing cultivation parameters or introducing specific enhancers.

### 3.3. Evaluation of the Immunochemical Properties of the Full-Length 10H10ch Antibody

**Figure 6:**
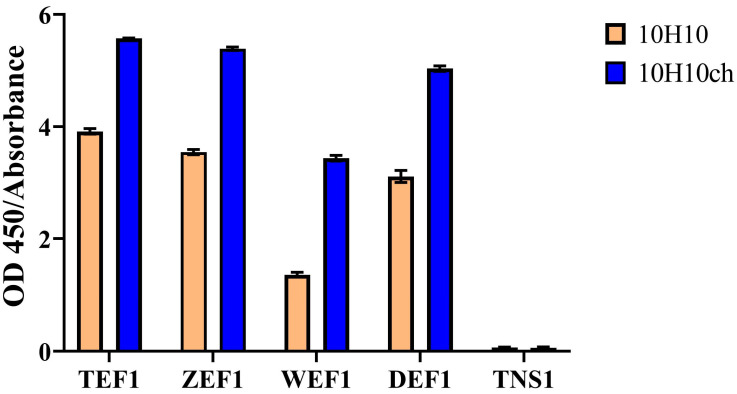
The results of the interaction of the recombinant 10H10ch and murine 10H10 antibodies with recombinant fragments of flavivirus envelope proteins are as follows: TEF1 represents the 1 + 2 domains of the E protein from tick-borne encephalitis virus; ZEF1 denotes the 1 + 2 domains of the E protein from Zika virus; WEF1 refers to the 1 + 2 domains of the E protein from West Nile virus; DEF1 comprises the 1 + 2 domains of the E protein from Dengue virus; and TNS1 indicates the non-structural protein 1 from tick-borne encephalitis virus.

Previous studies have shown that the mouse monoclonal antibody 10H10 interacts with high affinity, in the nanomolar range, with the E protein of tick-borne encephalitis virus (TBEV) as well as with E proteins of other flaviviruses [47]. The processes used to generate recombinant antibodies, including humanization and chimerization, may affect their functional activity [48,49]. Therefore, an immunochemical analysis of the produced antibody was performed using a panel of recombinant antigens. The antigen panel included fragments of E proteins from the flaviviruses TBEV, ZIKV, DENV, and WNV, as well as the non-structural protein 1 (NS1) of TBEV. The results are shown in Figure 6.

As expected, strong interaction signals were recorded for the TEF1, ZEF1, and DEF1 proteins, while no interaction was observed with the NS1 protein. These results are consistent with those obtained for the original mouse 10H10 antibody. The data confirm that the functional activity of the 10H10ch antibody was preserved after chimerization, demonstrating its ability to recognize the target epitope on the surface of flavivirus E proteins and indicating that the developed integration plasmid vector pVEAL3-10H10ch ensures the synthesis and secretion of full-length antibodies.

## 4. Conclusions

As part of the research program, an integration vector for the expression of recombinant proteins in mammalian cells was developed. This vector incorporates an artificial signaling sequence (176), which is a key feature of the construct. The vector was successfully used to produce a monoclonal antibody, designated 10H10, which exhibits specificity for the E surface protein of flaviviruses. The design of the vector includes several regulatory elements: the Gaussia luciferase signal sequence, IRES, P2A peptide, and furin cleavage site, which ensure the proper processing of the synthesized polypeptide and the assembly of the antibody’s heavy and light chains. The analysis of the culture fluid from the clones showed antibody production in over 95% of cases. The level of antibody production by adhesion culturing, without the optimization of conditions, was found to be 15 mg/L. These results confirm the promising potential of the proposed vector for the production of full-length antibodies and other recombinant proteins in cell systems.

## Data Availability

The original contributions presented in the study are included in the article; further inquiries can be directed to the corresponding author.

## Supplementary Materials

The following supporting information can be downloaded at: https://www.mdpi.com/article/10.3390/cimb46120868/s1, Figure S1: The complete nucleotide sequence of the integrative vector pVEAL3-10H10ch.

## References

[1] Hunter M., Yuan P., Vavilala D., Fox M. (2019). Optimization of Protein Expression in Mammalian Cells. Curr. Protoc. Protein Sci..

[2] Zhang J.H., Shan L.L., Liang F., Du C.Y., Li J.J. (2022). Strategies and Considerations for Improving Recombinant Antibody Production and Quality in Chinese Hamster Ovary Cells. Front. Bioeng. Biotechnol..

[3] Li Z.M., Fan Z.L., Wang X.Y., Wang T.Y. (2022). Factors Affecting the Expression of Recombinant Protein and Improvement Strategies in Chinese Hamster Ovary Cells. Front. Bioeng. Biotechnol..

[4] Carrara S.C., Fiebig D., Bogen J.P., Grzeschik J., Hock B., Kolmar H. (2021). Recombinant antibody production using a dual-promoter single plasmid system. Antibodies.

[5] Ahmadi M., Mahboudi F., Akbari Eidgahi M.R., Nasr R., Davami F. (2016). Evaluating the efficiency of phiC31 integrase-mediated monoclonal antibody expression in CHO cells. Biotechnol. Prog..

[6] Li Y.M., Wang M., Wang T.Y., Wei Y.G., Dou Y.Y. (2020). Effects of different 2A peptides on transgene expression mediated by tricistronic vectors in transfected CHO cells. Mol. Biol. Rep..

[7] Ebadat S., Ahmadi S., Ahmadi M., Nematpour F., Mahboudi F. (2017). Evaluating the efficiency of CHEF and CMV promoter with IRES and Furin/2A linker sequences for monoclonal antibody expression in CHO cells. PLoS ONE.

[8] Lin Y., Hung C.Y., Bhattacharya C., Nichols S., Xie J. (2018). An effective way of producing fully assembled antibody in transgenic tobacco plants by linking heavy and light chains via a self-cleaving 2a peptide. Front. Plant Sci..

[9] Ma W., Ma B., Ma J., Zhu R. (2023). RB1 5′UTR contains an IRES related to cell cycle control and cancer progression. Gene.

[10] Fitzgerald K.D., Semler B.L. (2009). Bridging IRES elements in mRNAs to the eukaryotic translation apparatus. Biochim. Biophys. Acta Gene Regul. Mech..

[11] Zhao J., Chen Z., Zhang M., Zou L., He S., Liu J., Wu J. (2024). DeepIRES: A hybrid deep learning model for accurate identification of internal ribosome entry sites in cellular and viral mRNAs. Brief. Bioinform..

[12] López-Ulloa B., Fuentes Y., Pizarro-Ortega M.S., López-Lastra M. (2022). RNA-Binding Proteins as Regulators of Internal Initiation of Viral mRNA Translation. Viruses.

[13] Andreev D.E., Niepmann M., Shatsky I.N. (2022). Elusive Trans-Acting Factors Which Operate with Type I (Poliovirus-like) IRES Elements. Int. J. Mol. Sci..

[14] Li D., Liu J., Sun L., Zhang J., Hou J. (2025). Developing polycistronic expression tool in Yarrowia lipolytica. Synth. Syst. Biotechnol..

[15] Ren Y., Lin Q., Berro J. (2023). 2A peptide from ERBV-1 efficiently separates endogenous protein domains in the fission yeast Schizosaccharomyces pombe. Micropubl. Biol..

[16] de Lima J.G.S., Lanza D.C.F. (2021). 2a and 2a-like sequences: Distribution in different virus species and applications in biotechnology. Viruses.

[17] Luke G.A., Ryan M.D. (2018). Using the 2A protein coexpression system: Multicistronic 2A vectors expressing gene(s) of interest and reporter proteins. Methods Mol. Biol..

[18] Sharma P., Yan F., Doronina V.A., Escuin-Ordinas H., Ryan M.D., Brown J.D. (2012). 2A peptides provide distinct solutions to driving stop-carry on translational recoding. Nucleic Acids Res..

[19] Luke G.A., Ross L.S., Lo Y., Wu H., Ryan M.D. (2024). Picornavirus Evolution: Genomes Encoding Multiple 2ANPGP Sequences—Biomedical and Biotechnological Utility. Viruses.

[20] Toporova V.A., Argentova V.V., Aliev T.K., Panina A.A., Dolgikh D.A., Kirpichnikov M.P. (2023). Optimization of recombinant antibody production based on the vector design and the level of metabolites for generation of Ig- producing stable cell lines. Genet. Eng. Biotechnol..

[21] Li J., Menzel C., Meier D., Zhang C., Dübel S., Jostock T. (2007). A comparative study of different vector designs for the mammalian expression of recombinant IgG antibodies. Immunol. Methods.

[22] Fallahee I., Hawiger D. (2024). Episomal Vectors for Stable Production of Recombinant Proteins and Engineered Antibodies. Antibodies.

[23] Kangro K., Roose E., Vandenbulcke A., Dekimpe C., Vanhoorelbeke K. (2022). Improvement of recombinant ADAMTS13 production through a more optimal signal peptide or an N-terminal fusion protein. Thromb. Haemost..

[24] Fu Y., Han Z., Cheng W., Niu S., Wang T., Wang X. (2024). Improvement strategies for transient gene expression in mammalian cells. Appl. Microbiol. Biotechnol..

[25] Yang C.H., Li H.C., Lo S.Y. (2024). Enhancing recombinant antibody yield in Chinese hamster ovary cells. Tzu Chi Med..

[26] Шaньшин Д.B., Щepбaкoв Д.H., Hecмeянoвa B.C., Иcaeвa А.А., Лoктeв B.Б., Пpoтoпoпoвa Е.B. Плaзмиднaя гeнeтичecкaя кoнcтpyкция pVEAL3-10H10ch, штaмм peкoмбинaнтнoй клeтoчнoй линии CHO-K1-10H10ch и химepнoe aнтитeлo 10H10ch пpoтив виpyca клeщeвoгo энцeфaлитa, пpoдyциpyeмoe yкaзaнным штaммoм клeтoчнoй линии CHO-K1-10H10ch. Пaтeнт нa изoбpeтeниe RU 2800471 C1, 21.07.2023. Заявка № 2022126714 oт 13.10.2022. https://elibrary.ru/item.asp?id=54232012.

[27] Li Y., Tian Z., Xu D., Wang X., Wang T. (2018). Construction strategies for developing expression vectors for recombinant monoclonal antibody production in CHO cells. Mol. Biol. Rep..

[28] Wang T.Y., Guo X. (2020). Expression vector cassette engineering for recombinant therapeutic production in mammalian cell systems. Appl. Microbiol. Biotechnol..

[29] Ho S.C., Bardor M., Feng H., Tong Y.W., Song Z., Yap M.G., Yang Y. (2012). IRES-mediated Tricistronic vectors for enhancing generation of high monoclonal antibody expressing CHO cell lines. Biotechnology.

[30] Shanshin D.V., Nesmeyanova V.S., Protopopova E.V., Shelemba A.A., Loktev V.B., Shcherbakov D.N. (2024). Preparation and Construction of Chimeric Humanized Broadly Reactive Antibody 10H10 to Protein E of Tick-Borne Encephalitis Virus. Bull. Exp. Biol. Med..

[31] Haбepeжнoв Д.C. (2021). Cpaвнитeльный aнaлиз пpoмoтopoв, иcпoльзyeмых для экcпpeccии тpaнcгeнoв в клeткaх млeкoпитaющих. Hayкocфepa.

[32] Csató-Kovács E., Salamon P., Fikó-Lászlo S., Kovács K., Koka A., András-Korodi M., Albert B. (2024). Development of a Mammalian Cell Line for Stable Production of Anti-PD-1. Antibodies.

[33] Bochkov Y.A., Palmenberg A.C. (2006). Translational efficiency of EMCV IRES in bicistronic vectors is dependent upon IRES sequence and gene location. Biotechniques.

[34] Wang X., Marchisio M.A. (2021). Synthetic polycistronic sequences in eukaryotes. Synth. Syst. Biotechnol..

[35] Van der Weken H., Cox E., Devriendt B. (2019). Rapid production of a chimeric antibody-antigen fusion protein based on 2A-peptide cleavage and green fluorescent protein expression in CHO cells. MAbs.

[36] Chung H., Buck L., Daris K., Welborn B., Luo Q., Wypych J. (2018). Investigation of the free heavy chain homodimers of a monoclonal antibody. Biotechnol. Prog..

[37] Borgoyakova M.B., Karpenko L.I., Rudometov A.P., Shanshin D.V., Ilyichev A.A. (2021). Immunogenic properties of the DNA construct encoding the receptor-binding domain of the SARS-CoV-2 spike protein. Mol. Biol..

[38] Ha T.K., Kim D., Kim C.L., Grav L.M., Lee G.M. (2022). Factors affecting the quality of therapeutic proteins in recombinant Chinese hamster ovary cell culture. Biotechnol. Adv..

[39] Hussain H., Patel T., Ozanne A.M., Vito D., Ellis M., Smales C.M. (2021). A comparative analysis of recombinant Fab and full-length antibody production in Chinese hamster ovary cells. Biotechnol. Bioeng..

[40] Choa JB D., Sasaki T., Kajiura H., Ikuta K., Fujiyama K., Misaki R. (2023). Effects of various disaccharide adaptations on recombinant IgA1 production in CHO-K1 suspension cells. Cytotechnology.

[41] Pirkalkhoran S., Grabowska W.R., Kashkoli H.H., Mirhassani R., Guiliano D., Dolphin C., Khalili H. (2023). Bioengineering of Antibody Fragments: Challenges and Opportunities. Bioengineering.

[42] Ochmann M.T., Ivics Z. (2021). Jumping ahead with sleeping beauty: Mechanistic insights into cut-and-paste transposition. Viruses.

[43] Ruf S., Symmons O., Uslu V.V., Dolle D., Hot C., Ettwiller L., Spitz F. (2011). Large-scale analysis of the regulatory architecture of the mouse genome with a transposon-associated sensor. Nat. Genet..

[44] Ivics Z., Izsvák Z., Medrano G., Chapman K.M., Hamra F.K. (2011). Sleeping Beauty transposon mutagenesis in rat spermatogonial stem cells. Nat. Protoc..

[45] Protopopova Y.V., Khusainova A.D., Konovalova S.N., Loktev V.B. (1996). Preparation and study of properties of anti-idiotypic antibodies, carrying hemagglutinating paratopes of tick-borne encephalitis virus on their surface. Vopr. Virusol..

[46] Frenzel A., Hust M., Schirrmann T. (2013). Expression of recombinant antibodies. Front. Immunol..

[47] Shanshin D.V., Borisevich S.S., Bondar A.A., Porozov Y.B., Shcherbakov D.N. (2022). Can Modern Molecular Modeling Methods Help Find the Area of Potential Vulnerability of Flaviviruses?. Int. J. Mol. Sci..

[48] Ormundo L.F., Barreto C.T., Tsuruta L.R. (2023). Development of Therapeutic Monoclonal Antibodies for Emerging Arbovirus Infections. Viruses.

[49] Kandari D., Bhatnagar R. (2023). Antibody engineering and its therapeutic applications. Int. Rev. Immunol..

