# Supplementary material for: Construction of an Integration Vector with a Chimeric Signal Peptide for the Expression of Monoclonal Antibodies in Mammalian Cells
Source: Curr Issues Mol Biol. 2024 Dec 22;46(12):14464–75. doi: 10.3390/cimb46120868 (PMC11674113; doi:10.3390/cimb46120868)
Supplement: Supplementary file 1 [file cimb-46-00868-s001.zip › cimb-3365331-supplementary.pdf]

Supplementary Figure S1. The complete nucleotide sequence of the integrative vector pVEAL3-10H10ch.

CGAAGAAAGGCCCACCCGTGAAGGTGAGCCAGTGAGTTGATTGCAGTCCAGTTACGCTGGA
GTCTGAGGCTCGTCCTGAATGGATCCCTATACAGTTGAAGTCGGAAGTTTACATACACTTAA
GTTGGAGTCATTAAAACTCGTTTTTCAACTACTCCACAAATTTCTTGTTAACAAACAATAGTT
TTGGCAAGTCAGTTAGGACATCTACTTTGTGCATGACACAAGTCATTTTTCCAACAATTGTTT
ACAGACAGATTATTTCACTTATAATTCACTGTATCACAATTCCAGTGGGTCAGAAGTTTACAT
ACACTAAGTTCGACTCCTCTGCAGAATGCGGCGATGTTTCGGTAAGGGGTCCGCTACTAGTT
ATTAATAGTAATCAATTACGGGGTCATTAGTTCATAGCCCATATATGGAGTTCCGCGTTACA
TAACTTACGGTAAATGGCCCGCCTGGCTGACCGCCCAACGACCCCCGCCCATTGACGTCAAT
AATGACGTATGTTCCCATAGTAACGCCAATAGGGACTTTCCATTGACGTCAATGGGTGGAGT
ATTTACGGTAAACTGCCCACTTGGCAGTACATCAAGTGTATCATATGCCAAGTACGCCCCCT
ATTGACGTCAATGACGGTAAATGGCCCGCCTGGCATTATGCCCAGTACATGACCTTATGGGA
CTTTCCTACTTGGCAGTACATCTACGTATTAGTCATCGCTATTACCATGGTGATGCGGTTTTG
GCAGTACATCAATGGGCGTGGATAGCGGTTTGACTCACGGGGATTTCCAAGTCTCCACCCCA
TTGACGTCAATGGGAGTTTGTTTTGGCACCAAAATCAACGGGACTTTCCAAAATGTCGTAAC
AACTCCGCCCCATTGACGCAAATGGGCGGTAGGCGTGTACGGTGGGAGGTCTATATAAGCA
GAGCTCTCTGGCTAACTAGAGAACCCACTGCTTACTGGCTTATCGAAATTAATACGACTCAC
TATAGGGAGACCCAAGCTGGCTAGCACCATGATGCGGACCCTGATCCTGGCTGTGCTGCTGG
TGTACTTCTGTGCCACCGTGCACTGCTCCGACATTGTGCTGACCCAGACTCCACTCACTTTGT
CGGTTACCATTGGACAGCCAGCCTCCATCTCTTGCAAGTCAAGTCAGAGCCTCTTAGATAGT
GATGGAAAGACATATTTGAATTGGTTGTTACAGAGGCCAGGCCAGTCTCCAAAGCGCCTAAT
CTATCTGGTGTCTAAATTGGACTCTGGAGTCCCTGACAGGTTCACTGGCGGTGGATCAGGGA
CAGATTTCACACTGAAAATCAGCAGAGTGGAGGCTGAGGATTTGGGAGTTTATTATTGCTGG
CAAGGTACACATTTTCCTCAGACGTTCGGTGGAGGCACCAAGCTGGAAATCAAACGTACGGT
GGCTGCACCATCTGTCTTCATCTTCCCGCCATCTGATGAGCAGTTGAAATCTGGAACTGCCTC
TGTTGTGTGCCTGCTGAATAACTTCTATCCCAGAGAGGCCAAAGTACAGTGGAAGGTGGATA
ACGCCCTCCAATCGGGTAACTCCCAGGAGAGTGTCACAGAGCAGGACAGCAAGGACAGCAC
CTACAGCCTCAGCAGCACCCTGACGCTGAGCAAAGCAGACTACGAGAAACACAAAGTCTAC
GCCTGCGAAGTCACCCATCAGGGCCTGAGCTCGCCCGTCACAAAGAGCTTCAACAGGGGAG
AGTGTAGACGGAAGAGAGCCACCAACTTTAGTCTGCTGAAGCAGGCCGGCGACGTGGAAGA
GAATCCTGGACCTATGGGAGTGAAGGTGCTGTTCGCCCTGATCTGTATTGCCGTGGCCGAAG
CTTCTGTGAAGCTGGAGGAGTCTGGGGGAGGCTTAGTGAAGCCTGGAGGGTCCCTGAAACT
CTCCTGTGCAGCCTCTGGATTCAGTTTCAGTGACTATTACATGTATTGGGTTCGTCAGACTCC
GGAAAAGAGGCTGGAGTGGGTCGCAACCATTAGTGATGGTGGTAGTCACACCTCCTATCGA
GACAGTGTGAAGGGGCGATTTACCATCTCCAGAGACAATGGCAAGAACAACCTGTACCTGC
AAATGAGCAGTCTGAAGTCTGAGGACACAGCCATGTATTACTGTGTAAGAGGTGCTTACTGG
GGCCAAGGGACTCTGGTCACTGTCTCTTCTGCCAGCACCAAGGGGCCAAGCGTCTTCCCCCT
GGCACCCTCCTCCAAGAGCACCTCTGGGGGCACAGCGGCCCTGGGCTGCCTGGTCAAGGACT
ACTTCCCCGAACCGGTGACGGTGTCGTGGAACTCAGGCGCCCTGACCAGCGGCGTGCACACC
TTCCCGGCTGTCCTACAGTCCTCAGGACTCTACTCCCTCAGCAGCGTGGTGACCGTGCCCTCC
AGCAGCTTGGGCACCCAGACCTACATCTGCAACGTGAATCACAAGCCCAGCAACACCAAGG
TGGACAAGAAAGTTGAGCCCAAATCTTGTGACAAAACTCACACATGCCCACCGTGCCCAGC
ACCTGAACTCCTGGGGGGACCGTCAGTCTTCCTCTTCCCCCCAAAACCCAAGGACACCCTCT
ACATCACCCGGGAACCTGAGGTCACATGCGTGGTGGTGGACGTGAGCCACGAAGACCCTGA
GGTCAAGTTCAACTGGTACGTGGACGGCGTGGAGGTGCATAATGCCAAGACAAAGCCGCGG
GAGGAGCAGTACAACAGCACGTACCGTGTGGTCAGCGTCCTCACCGTCCTGCACCAGGACT
GGCTGAATGGCAAGGAGTACAAGTGCAAGGTCTCCAACAAAGCCCTCCCAGCCCCCATCGC
GAAAACCATCTCCAAAGCCAAAGGGCAGCCCCGAGAACCACAGGTGTACACCCTGCCCCCA
TCCCGGGAGGAGATGACCAAGAACCAGGTCAGCCTGACCTGCCTGGTCAAAGGCTTCTATCC
CAGCGACATCGCCGTGGAGTGGGAGAGCAATGGGCAGCCGGAGAACAACTACAAGACCAC
GCCTCCCGTGCTGGACTCCGACGGCTCCTTCTTCCTCTACAGCAAGCTCACCGTGGACAAGA
GCAGGTGGCAGCAGGGGAACGTCTTCTCATGCTCCGTGATGCATGAGGCTCTGAAGTTCCAC

TACACGCAGAAGAGCCTCTCCCTGTCTCCGGGTAAATGAGTCGACCGAGCGGTTCCCGCCCC
TCTCCCTCCCCCCCCCCTAACGTTACTGGCCGAAGCCGCTTGGAATAAGGCCGGTGTGCGTTT
GTCTATATGTTATTTTCCACCATATTGCCGTCTTTTGGCAATGTGAGGGCCCGGAAACCTGGC
CCTGTCTTCTTGACGAGCATTCCTAGGGGTCTTTCCCCTCTCGCCAAAGGAATGCAAGGTCTG
TTGAATGTCGTGAAGGAAGCAGTTCCTCTGGAAGCTTCTTGAAGACAAACAACGTCTGTAGC
GACCCTTTGCAGGCAGCGGAACCCCCCACCTGGCGACAGGTGCCTCTGCGGCCAAAAGCCA
CGTGTATAAGATACACCTGCAAAGGCGGCACAACCCCAGTGCCACGTTGTGAGTTGGATAGT
TGTGGAAAGAGTCAAATGGCTCACCTCAAGCGTATTCAACAAGGGGCTGAAGGATGCCCAG
AAGGTACCCCATTGTATGGGATCTGATCTGGGGCCTCGGTGCACATGCTTTACATGTGTTTA
GTCGAGGTTAAAAAACGTCTAGGCCCCCCGAACCACGGGGACGTGGTTTTCCTTTGAAAAAC
ACGATGATAATATGGCCACAACCATGACCGAGTACAAGCCCACGGTGCGCCTCGCCACCCG
CGACGACGTCCCCAGGGCCGTACGCACCCTCGCCGCCGCGTTCGCCGACTACCCCGCCACGC
GCCACACCGTCGATCCGGACCGCCACATCGAGCGGGTCACCGAGCTGCAAGAACTCTTCCTC
ACGCGCGTCGGGCTCGACATCGGCAAGGTGTGGGTCGCGGACGACGGCGCCGCGGTGGCGG
TCTGGACCACGCCGGAGAGCGTCGAAGCGGGGGCGGTGTTCGCCGAGATCGGCCCGCGCAT
GGCCGAGTTGAGCGGTTCCCGGCTGGCCGCGCAGCAACAGATGGAAGGCCTCCTGGCGCCG
CACCGGCCCAAGGAGCCCGCGTGGTTCCTGGCCACCGTCGGCGTCTCGCCCGACCACCAGGG
CAAGGGTCTGGGCAGCGCCGTCGTGCTCCCCGGAGTGGAGGCGGCCGAGCGCGCCGGGGTG
CCCGCCTTCCTGGAGACCTCCGCGCCCCGCAACCTCCCCTTCTACGAGCGGCTCGGCTTCACC
GTCACCGCCGACGTCGAGGTGCCCGAAGGACCGCGCACCTGGTGCATGACCCGCAAGCCCG
GTGCCTGATTCGCATATGGGTTAATGCTTCGAGCAGACATGATAAGATACATTGATGAGTTT
GGACAAACCACAACTAGAATGCAGTGAAAAAAATGCTTTATTTGTGAAATTTGTGATGCTAT
TGCTTTATTTGTAACCATTATAAGCTGCAATAAACAAGTTCCTCGACCTCTAGCTAGAGCTAC
TCGGGACCCCTTACCGAAACATCGCCGCATTCTGCAGAGGAGTCGAGTGTATGTAAACTTCT
GACCCACTGGGAATGTGATGAAAGAAATAAAAGCTGAAATGAATCATTCTCTCTACTATTAT
TCTGATATTTCACATTCTTAAAATAAAGTGGTGATCCTAACTGACCTAAGACAGGGAATTTTT
ACTAGGATTAAATGTCAGGAATTGTGAAAAAGTGAGTTTAAATGTATTTGGCTAAGGTGTAT
GTAAACTTCCGACTTCAACTGTATAGGGATCCGCTCAATACTGACCATTTAAATCATACCTG
ACCTCCATAGCAGAAAGTCAAAAGCCTCCGACCGGAGGCTTTTGACTTGATCGGCACGTAAG
AGGTTCCAACTTTCACCATAATGAAATAAGATCACTACCGGGCGTATTTTTTGAGTTATCGA
GATTTTCAGGAGCTAAGGAAGCTAAAATGAGCCATATTCAACGGGAAACGTCTTGCTCGAG
GCCGCGATTAAATTCCAACATGGATGCTGATTTATATGGGTATAAATGGGCTCGCGATAATG
TCGGGCAATCAGGTGCGACAATCTATCGATTGTATGGGAAGCCCGATGCGCCAGAGTTGTTT
CTGAAACATGGCAAAGGTAGCGTTGCCAATGATGTTACAGATGAGATGGTCAGGCTAAACT
GGCTGACGGAATTTATGCCTCTTCCGACCATCAAGCATTTTATCCGTACTCCTGATGATGCAT
GGTTACTCACCACTGCGATCCCAGGGAAAACAGCATTCCAGGTATTAGAAGAATATCCTGAT
TCAGGTGAAAATATTGTTGATGCGCTGGCAGTGTTCCTGCGCCGGTTGCATTCGATTCCTGTT
TGTAATTGTCCTTTTAACGGCGATCGCGTATTTCGTCTGGCTCAGGCGCAATCACGAATGAAT
AACGGTTTGGTTGGTGCGAGTGATTTTGATGACGAGCGTAATGGCTGGCCTGTTGAACAAGT
CTGGAAAGAAATGCATAAGCTTTTGCCATTCTCACCGGATTCAGTCGTCACTCATGGTGATTT
CTCACTTGATAACCTTATTTTTGACGAGGGGAAATTAATAGGTTGTATTGATGTTGGACGAG
TCGGAATCGCAGACCGATACCAGGATCTTGCCATCCTATGGAACTGCCTCGGTGAGTTTTCT
CCTTCATTACAGAAACGGCTTTTTCAAAAATATGGTATTGATAATCCTGATATGAATAAATT
GCAGTTTCACTTGATGCTCGATGAGTTTTTCTAATGAGGGCCCAAATGTAATCACCTGGCTCA
CCTTCGGGTGGGCCTTTCTGCGTTGCTGGCGTTTTTCCATAGGCTCCGCCCCCCTGACGAGCA
TCACAAAAATCGATGCTCAAGTCAGAGGTGGCGAAACCCGACAGGACTATAAAGATACCAG
GCGTTTCCCCCTGGAAGCTCCCTCGTGCGCTCTCCTGTTCCGACCCTGCCGCTTACCGGATAC
CTGTCCGCCTTTCTCCCTTCGGGAAGCGTGGCGCTTTCTCATAGCTCACGCTGTAGGTATCTC
AGTTCGGTGTAGGTCGTTCGCTCCAAGCTGGGCTGTGTGCACGAACCCCCCGTTCAGCCCGA
CCGCTGCGCCTTATCCGGTAACTATCGTCTTGAGTCCAACCCGGTAAGACACGACTTATCGC
CACTGGCAGCAGCCACTGGTAACAGGATTAGCAGAGCGAGGTATGTAGGCGGTGCTACAGA
GTTCTTGAAGTGGTGGCCTAACTACGGCTACACTAGAAGAACAGTATTTGGTATCTGCGCTC
TGCTGAAGCCAGTTACCTCGGAAAAAGAGTTGGTAGCTCTTGATCCGGCAAACAAACCACC

GCTGGTAGCGGTGGTTTTTTTGTTTGCAAGCAGCAGATTACGCGCAGAAAAAAAGGATCTCA
AGAAGATCCTTTGATTTTCTAC

– CMV promoter region

– 176 – nucleotide sequence encoding a hybrid signal peptide from luciferase (Cypridina noctiluca) and fibroin (Dendrolimus spectabilis), facilitating protein export from the cell

– Expression cassette

– EMCV IRES – internal ribosome entry site

– PuroR – nucleotide sequence encoding resistance to the antibiotic puromycin

– SV40 poly (A) signal – nucleotide sequence stabilizing mRNA transcripts through polyadenylation
